# Ruthenium(ii) polypyridyl complexes and the DNA damage response: mechanisms and therapeutic implications

**DOI:** 10.1039/d6md00154h

**Published:** 2026-05-06

**Authors:** Martin R. Gill

**Affiliations:** a Department of Chemistry, Faculty of Science and Engineering, Swansea University UK m.r.gill@swansea.ac.uk

## Abstract

Ruthenium(ii) polypyridyl complexes (RPCs) have generated substantial interest due to their biomolecular binding capabilities, favourable photophysical properties, and anticancer activity. DNA is widely reported as a target for RPCs, and their recent development as photosensitisers for photodynamic therapy further emphasises DNA damage as a key biological outcome. The aim of this review is to highlight recent studies in the design of RPCs as pharmacological DNA-targeting agents and describe what is known about their impact on the DNA damage response (DDR). This, in turn, provides insight into the nature of the DNA lesions induced by these complexes. The relationship between binding mode, activation of specific DDR pathways, and resultant cell fate in human cancer cell lines is examined and, where appropriate, placed in a therapeutic context. Implications for enhancing cancer selectivity, including the use of RPCs alongside DDR inhibitors in combination strategies, as well as associated safety considerations, are discussed.

## Abstract 2

This review summarises the design of ruthenium(ii) polypyridyl complexes (RPCs) as pharmacological DNA-targeting agents, linking binding mode, DDR pathway activation, and cancer cell fate.

## Graphical abstract

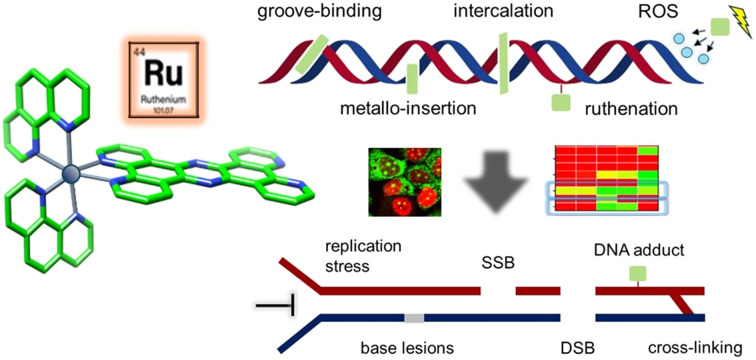

## Introduction

Cancer remains a substantial health and economic challenge in society. In the UK, there are more than 386 000 new cancer cases and 168 000 cancer deaths every year, where it represents the leading cause of mortality.^1^ Incidence rates are rising, resulting in a predicted 506,000 new cases by 2038-2040, although mortality rates are projected to fall by 6% in this same time period.^1^ Modelling by the Organisation for Economic Co-operation and Development (OECD) estimates that the UK's health expenditure on cancer is currently £14.4 billion per year and, without action, predicts it will increase by 52% *per capita* by 2050.^2^

Since the conversion of the nitrogen mustards from chemical warfare agents to chemotherapeutics in the 1940s, DNA-damaging chemotherapy has remained a cornerstone of cancer treatment.^3^ The basis for DNA damaging chemotherapy is that cancer cells are more sensitive to DNA damage based on their inherent genomic instability and high rates of replication.^4^ Several classes of DNA damaging chemotherapeutics have been developed, including alkylating agents, antimetabolites, crosslinking agents, topoisomerase inhibitors and antibiotics. Treatment is often limited by side-effects, which can include long-term genomic damage to healthy cells. However, the wide variety of chemotherapeutic drugs available possess individual variations in activity, which depends on the precise form of DNA lesion produced in addition to individual pharmacokinetic and pharmacodynamic effects.^5^

Complementary to this work, significant advances in elucidating the cellular response to DNA damage have been made, describing a network of signalling pathways collectively known as the DDR. Investigation of the DDR response to DNA-damaging therapeutics has provided information on both the nature of lesion(s) generated and the differing responses of cancer types or non-cancer cells.^6^ For example, cancers with up-regulated DDR pathways can show an increased ability to repair DNA damage induced by chemotherapy and demonstrate resistance, while a deficiency in a key DDR protein may result in a cancer being more susceptible to therapy. Along with advances in genomics and early detection, these mechanistic insights have enabled increasingly personalised patient treatment.^7^ Furthermore, based on the concept that cancers with a defect in a particular DDR pathway can be highly sensitive to inhibition of a complementary pathway (a concept known as synthetic lethality), inhibitors for specific DDR proteins have emerged as novel therapeutic agents.^8^ The best clinical example of this is the use of PARP inhibitors towards breast cancers deficient in BRCA1 or BRCA2 function, work that has resulted in four PARP inhibitors now approved for clinical use.^9^ DDR inhibitors are additionally generating considerable interest in combination therapy roles where the addition of a DDR inhibitor has the potential to increase DNA damage generated by chemo- or radiotherapy.^10^ This means that once the nature of DDR signalling in response to a DNA-damaging agent has been established, there will be a strong mechanistic basis to synergise with pathway-relevant DDR inhibitors based upon complementary mechanisms of action. Such drug synergy has been examined in several clinical trials and appears to hold greatest promise when directed at drug-resistant or aggressive cancers, or those harbouring specific molecular defects.^11^

Although the majority of small molecule DNA-targeting therapeutics are organic compounds, employing transition metal chemistry to design pharmacophores for medicinal chemistry offers numerous attractive aspects.^12,13^ The platinum drugs remain the most successful clinical examples, where the fortuitous discovery of the anti-proliferative effects of cisplatin (*cis*-diamminedichloroplatinum(ii)) and its subsequent translation to anti-cancer drug spurred interest in metallodrugs and DNA-binding metal complexes. Mechanistically, cisplatin acts to form platinum–DNA adducts through Pt(ii) coordinating the N7 atoms of the imidazole rings of guanine and adenine. Such monofunctional DNA adducts further react to produce inter- or intrastrand cross-links that block DNA replication and/or transcription.^14^ Cisplatin-induced DNA damage activates multiple components of the DDR, including the ATM/Chk2 (ATM = ataxia-telangiectasia mutated), ATR/Chk1 (ATM and Rad3-related) and poly(ADP-ribose) polymerase (PARP) pathways.^14^ This has led to the development of numerous synergistic combinations of platinum drugs with DDR inhibitors, most notably PARP and ATR inhibitors, several of which are now under assessment in clinical trials.^15^

**Fig. 1:**
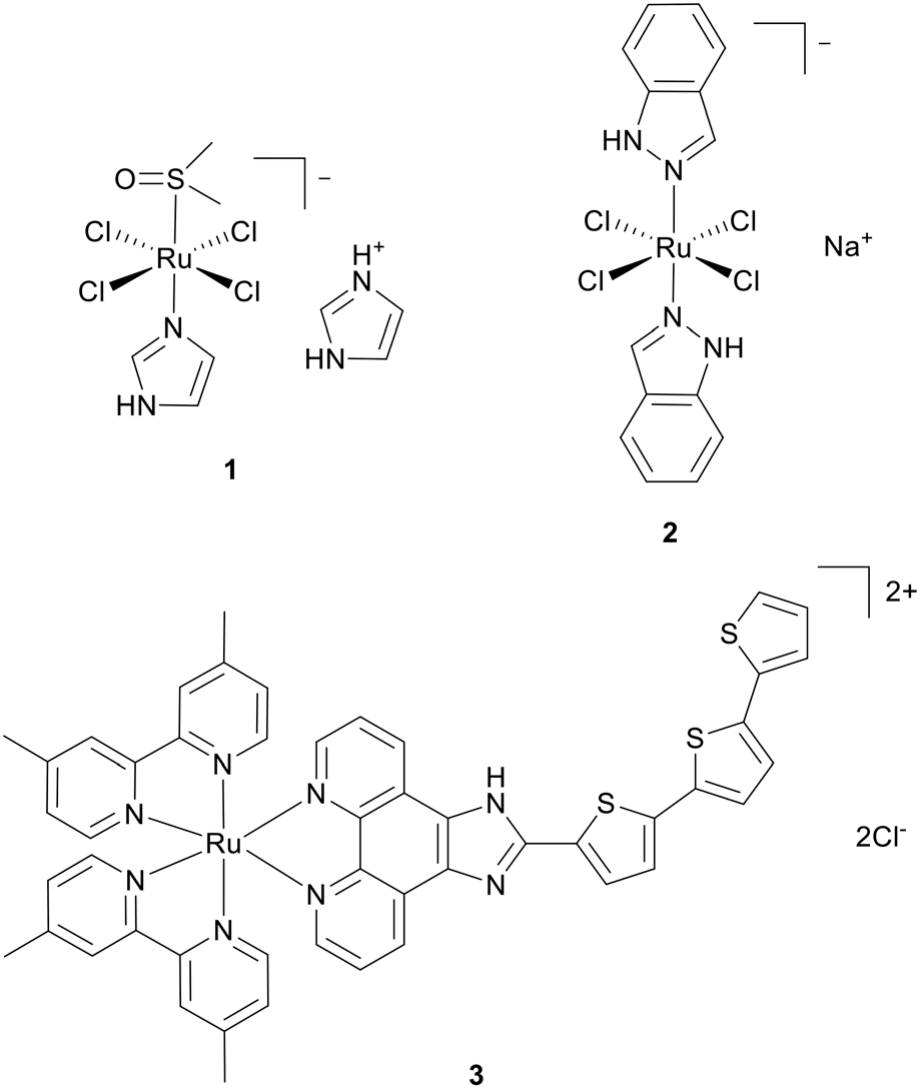
Structures of ruthenium-containing drugs that have been in clinical trials. 1 = NAMI-A, 2 = NKP1339, 3 = TLD-1433 aka Ruvidar™.

The success of cisplatin has inspired a vast quantity of work into metal-based compounds in cancer research, where candidates aim to improve upon cancer selectivity and circumvent acquired or intrinsic platinum resistance. This is perhaps best demonstrated by work on ruthenium(ii) and ruthenium(iii) complexes where three complexes have entered clinical trials to date: NAMI-A (1), KP1019 (NKP1339 as the sodium salt, aka BOLD-100; 2) and TLD-1433 (Ruvidar™; 3) (Fig. 1).^16–18^ Mechanistically, NAMI-A targets the extracellular matrix (ECM) and prevents metastasis,^19^ BOLD-100 has a multimodal mechanism, most recently its status as an anti-Warburg drug was reported,^20^ while TLD-1433 is a photosensitizer for use in photodynamic therapy (PDT).^18^

This review focuses on the design of RPCs as DNA-targeting agents. Examples provided summarise current knowledge of their effects on the DNA damage response (DDR) and resultant cellular fate(s) in human cells, particularly cancer cell lines. While numerous RPCs have been characterised for their cell-free DNA binding properties alongside cytotoxic or cytostatic potencies,^21,22^ subsequent evaluation of DDR activation provides a critical means of target validation, as cellular responses reveal the nature of the DNA lesions induced and establish links between *in vitro* binding and biological activity. Considering the structural diversity presented by polypyridyl ligands, these insights can aid the rational design of therapeutic candidates, understand cancer selectivity and guide synergistic drug combinations. Of course, DNA is by no means the sole target for RPCs and another review that covers the breadth of their design principals for a wider range of molecular targets is recommended to assess the full capabilities of RPCs within medicine.^23^

## Ruthenium(ii) polypyridyl complexes as DNA-target agents

### DNA binding modes and early work

Binding modes by which small molecules can interact with DNA include covalent or coordination bond formation with the bases or sugar-phosphate backbone or reversible interactions such as electrostatic, groove-binding, intercalation and insertion (reviewed in detail in ref. 24). Binding modes are non-exclusive and DNA-binding molecules may demonstrate several. For example, the DNA dye DAPI, which was originally viewed as a potential anti-cancer drug candidate, is both a DNA intercalator and groove-binder depending on the precise sequence employed.^25^

**Fig. 2:**
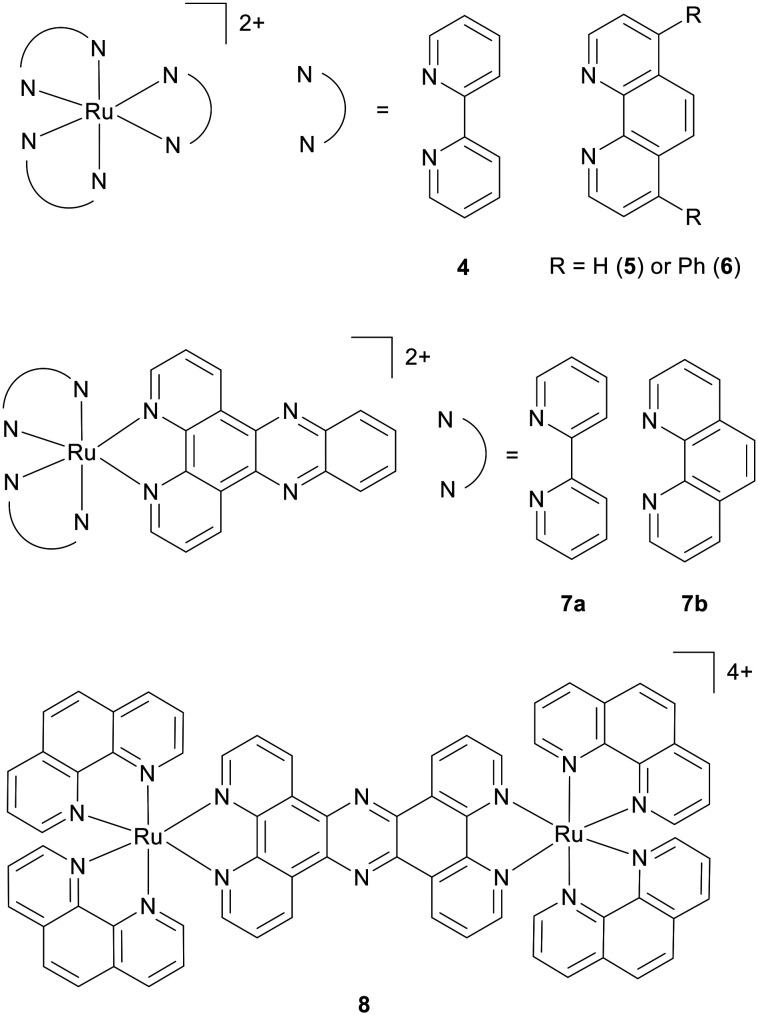
Structure of early DNA-binding polypyridyl complexes (RPCs) 4–7b and DNA imaging agent 8.

Ru(ii) complexes employing polypyridyl ligands have several advantageous properties to bind DNA, including octahedral geometry that can support multi-functionality, tuneable redox and photophysical properties, and the ability to emit from long-lived metal-to-ligand (MLCT) charge transfer states.^22^ DNA binding interactions of early candidates such as [Ru(bpy)_3_]^2+^ (bpy = 2,2′-bipyridine; 4), [Ru(phen)_3_]^2+^ (phen = 1,10-phenanthroline; 5) and [Ru(DIP)_3_]^2+^ (DIP = 4,7-diphenyl-1,10-phenanthroline; 6) (Fig. 2) were dominated by electrostatic interactions^26,27^ and required extended polypyridyl ligands such as dppz (dipyrido[2-*a*:2′,3′-*c*]phenazine) to achieve more advanced reversible binding modes such as intercalation.^28^ The discovery that the prototypical RPC DNA-binding complexes [Ru(bpy)_2_(dppz)]^2+^ (7a) and [Ru(phen)_2_(dppz)]^2+^ (7b) (Fig. 2) exhibit high DNA binding affinities accompanied by a pronounced increase in MLCT luminescence intensity upon DNA binding generated substantial interest in the field.^28,29^ The behaviour of RPCs as “light-switch” molecules provides a convenient means of measuring binding to a range of DNA sequences and structures, while also attracting interest for development as DNA probes and imaging agents.^30^ Based on these early studies, and the synthetic pathways utilised (see ref. 31–33 and references within), molecules are typically heteroleptic [Ru(*N*^*N*)_2_(*N*′*^N*′)]^2+^ complexes designed to feature a DNA-interactive ditopic polypyridyl ligand, most commonly an intercalating ligand, and ancillary ligands that can fine-tune affinity, selectivity or photophysical properties. Such complexes contain a chiral Ru(ii) centre and, unless stated otherwise, complexes described in biological studies below were used as racemic mixtures. One strength of utilising Ru(ii) chemistry is the capacity to design complexes with multifunctional properties. For example, a single molecule can be engineered to exhibit multiple DNA-binding modes, incorporate ligands with complementary bioactivity, or display stimuli-responsive behaviour.

### Cellular uptake and intracellular localisation

Along with basic cytotoxicity characterisation, cellular localisation studies and organelle targeting are often performed at an early stage of investigation, where high accumulation in the nucleus (or mitochondria) can serve as a starting point to indicate a DNA-targeting mechanism of action for a cytotoxic or cytostatic molecule. The ability of RPCs to phosphoresce in the visible region from long-lived ^3^MLCT states has enabled the examination of cellular internalisation by fluorescence, confocal and lifetime microscopies. This has been particularly useful for “light switch” complexes, where such luminescence provides evidence of intracellular DNA binding.^34–36^ As would be expected considering the breadth of chemical diversity available, a range of uptake mechanisms for RPCs have been described in a complex-specific manner, including passive diffusion,^37,38^ endocytosis^39,40^ and other forms of active transport.^41^ In addition to nuclear targeting, numerous mitochondrial targeting RPCs have also been described.^42^ This outcome that can be explained as hydrophobic cations have a natural affinity for the negative electric potential across the inner membranes of these organelles,^43^ and reflects the influence of early work promoting the design of RPCs for increased hydrophobicity to improve cellular uptake.^37^ It is also the case that molecules may target both the nucleus and mitochondria, and even that specific organelle localisation may be controlled by uptake pathway.^44^

Whilst early studies relied upon convenient imaging techniques such as confocal laser scanning microscopy (CLSM) heavily, quantitative techniques such as inductively coupled plasma mass spectroscopy (ICP-MS) in combination with cellular fractionation have emerged to further improve the ability to examine sub-cellular localisation. As these techniques do not rely upon visualisation of the molecule by microscopy, this has naturally promoted greater interest in non-luminescent RPCs compared to early work that focused on MLCT-emissive complexes. It is also worth stating that organelle localisation by itself does not necessarily equate to functional activity; a complex may be present in the nucleus at low abundance yet still exert a significant effect on DNA structure and/or function. In such cases, DNA would still be described as the pharmacological target of the complex. Conversely, high nuclear accumulation does not necessarily translate to a DNA-dependent mechanism. Take, for instance, the groove-binding imaging agent [(Ru(phen)_2_)_2_(tpphz)]^4+^ (tpphz = tetrapyrido[3,2-*a*:2′,3′-*c*:3″,2″-*h*:2‴,3‴-*j*]phenazine; 8, Fig. 2):^41^ in addition to its nuclear DNA imaging capacity, longer term exposure showed a potent impact on cell proliferation (72 h IC_50_ of 6 μM towards MCF7 breast cancer cells; the same potency as cisplatin). However, despite the high nuclear accumulation evidenced in multiple techniques, including CLSM, TEM and ICP-MS, mechanistic studies indicated no DNA damage pathway activation. Instead, 8 inhibits actin polymerisation, disrupting cytoskeleton assembly and blocking cytokinesis as a result.^45^

### Cellular DNA damage

**Fig. 3:**
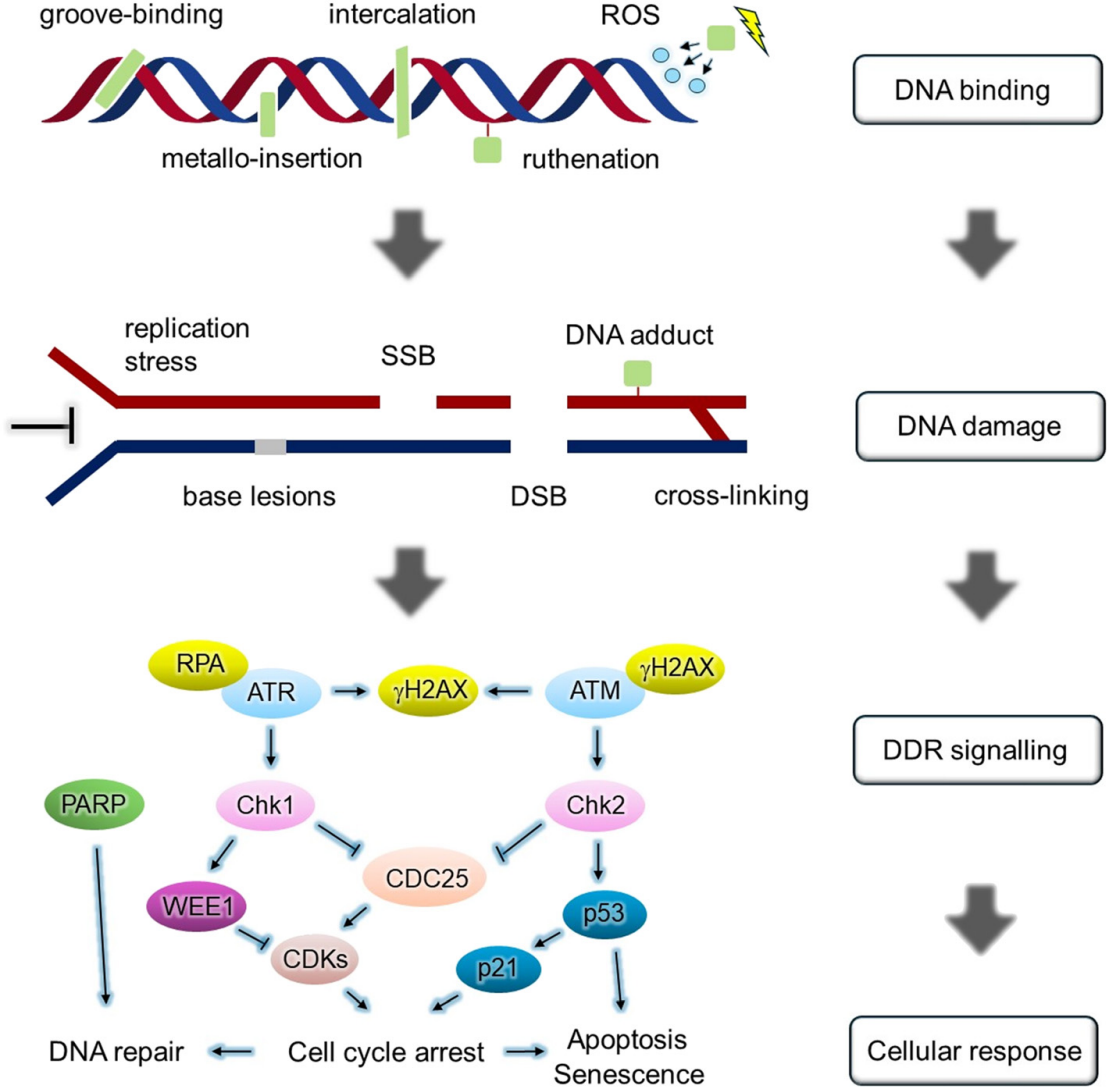
Schematic diagram of relationship between DNA binding modes and DNA damage response.

DNA damage by small molecules can be achieved by several mechanisms, including direct binding, cross-link formation, modifications such as alkylation and adduct formation, the generation of reactive oxygen species (ROS), inhibition of replication. Such interactions can lead to genomic damage in the form of strand cleavage as single-strand breaks (SSB), double-strand breaks (DSBs) or formation of other lesions. Binding can additionally impact DNA structure, such as the lengthening and unwinding of the helix upon intercalation or even distinct kinking of DNA by semi-intercalation.^46^ Indirect mechanisms for DNA damage also exist, such as ROS generation, where species such as O_2_^−^, H_2_O_2_ and ·OH can oxidise DNA bases and damage the sugar backbone, leading to strand breaks.^47^ When such damage is detected by the cell, cell cycle progress can be halted and DNA damage repair mechanisms activated. However, if such damage is not repaired, this can trigger cell death pathways, most commonly apoptosis or senescence (Fig. 3).^7^

A variety of DDR pathways operate to deal with different forms of DNA damage. These pathways can be categorised based on the nature of DNA lesion generated and the DNA repair mechanism involved. Comprehensive reviews on the underlying molecular biology are available elsewhere;^48,49^ briefly, and for relevance here, the protein kinases ATM and ATR function as two master upstream regulators: ATM is activated in response to DSB damage while ATR is activated by replication stress and SSB damage. ATR and ATM phosphorylate and activate various target proteins, including the cell cycle checkpoints Chk1 and Chk2, leading to cell-cycle arrest through inhibition of cyclin-dependent kinases or activation of p53. Other key DDR mediators include histone H2AX, a substrate of both ATM and ATR (primarily ATM) that becomes phosphorylated at Ser139 position (γH2AX) in response to DSBs and other lesions; WEE1, a cell cycle checkpoint kinase that controls entry into mitosis; and PARP-1, which is involved in the detection and repair of SSBs.^50,51^ A simplified schematic of DDR signalling is shown in Fig. 3.

## Double-strand break generators

### Photosensitizers

**Fig. 4:**
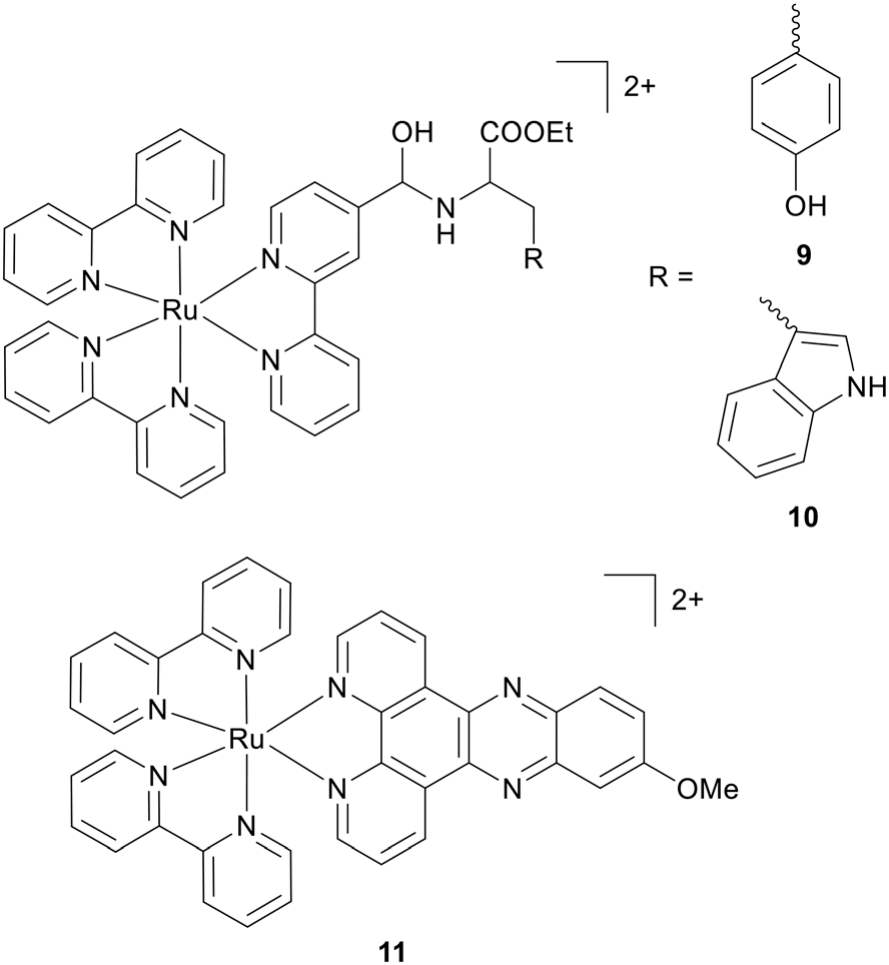
Structures of DSB-generating RPC photosensitizers 9–11.

Photodynamic therapy (PDT), the combination of light and a photosensitizer to generate localised cell death, relies upon ROS-mediated DNA damage. The ability of RPCs to generate singlet oxygen or other ROS upon exposure to light courtesy of excitation into long-lived, triplet MLCT states has seen a resurgence of interest in RPCs as photosensitizers in the last decade, and resulted in one candidate – TLD1433 – entering phase II clinical trials for bladder cancer.^18,52,53^ The cell-free plasmid pBR322 DNA assay has been particularly effective in isolating RPCs able to photocleave DNA, where unwinding of the supercoiled form provides an indication of SSB and DSB frequency. This assay can also establish the mechanism of DNA cleavage by employing selective reactive oxygen species quenchers (*e.g.* for hydrogen peroxide, singlet oxygen or hydroxyl radical *etc.*).^54^ In cells, it is well-documented that reactive oxygen species (ROS) induce DNA damage primarily by DSB formation and base oxidation, where genotoxic DSBs represent the most cytotoxic adducts and trigger cell-cycle arrest and apoptosis if unrepaired.^55^ In addition to this, oxidised bases can present a barrier to replication and ROS accumulation can also induce mitochondrial DNA lesions and impact mitochondrial function.^55^ Despite the large volume of work exploring RPCs as photosensitizers, few studies examine the cellular DNA damage response accompanying phototoxicity. In one example, Ramu *et al.* reported high levels of light-dependent intracellular DNA cleavage in A549 human lung adenocarcinoma and Hct116 human colorectal carcinoma cells in response to treatment with tyrosine or tryptophan functionalised RPCs (9 and 10, Fig. 4), as determined by comet assay.^56^ Phototoxicity was confirmed in spheroid models and inhibitor studies provided evidence it was due to ^1^O_2_ generation. Interestingly, while both molecules bind DNA in cell-free conditions by reversible binding, uptake studies revealed mitochondria and membrane-accumulation accompanied by no evidence of nuclear uptake, showing how the latter is not a pre-requirement for light-activated DNA damage.

Another example is presented by Pierroz *et al.* who examined the UV-light mediated cytotoxic action of [Ru(bpy)_2_-dppz-7-methoxy][PF_6_]_2_ (11, Fig. 4), a derivative of the prototypical RPC intercalator 7, in U2OS osteosarcoma cells.^57^ Nuclear localisation in U2OS, MCF7, and CAL33 cancer cells along with normal retinal epithelial cell line RPE-1 was described and alkaline comet assay and γH2AX expression provided evidence of DSB formation in a light-assisted manner. This work featured an intriguing dual mechanism of cell death: interphase cells experienced cell death by an ER-stress pathway while a mitotic block was additionally observed. These studies serve to illustrate both the underexplored biological responses to RPC-induced phototoxicity and the complexity of cellular responses to ROS, where there evidently remains much to be discovered. However, as Monro *et al.* noted, these aspects will additionally be impacted by variables associated with light parameters and dosimetry.^18^

### Non-photosensitizers

**Fig. 5:**
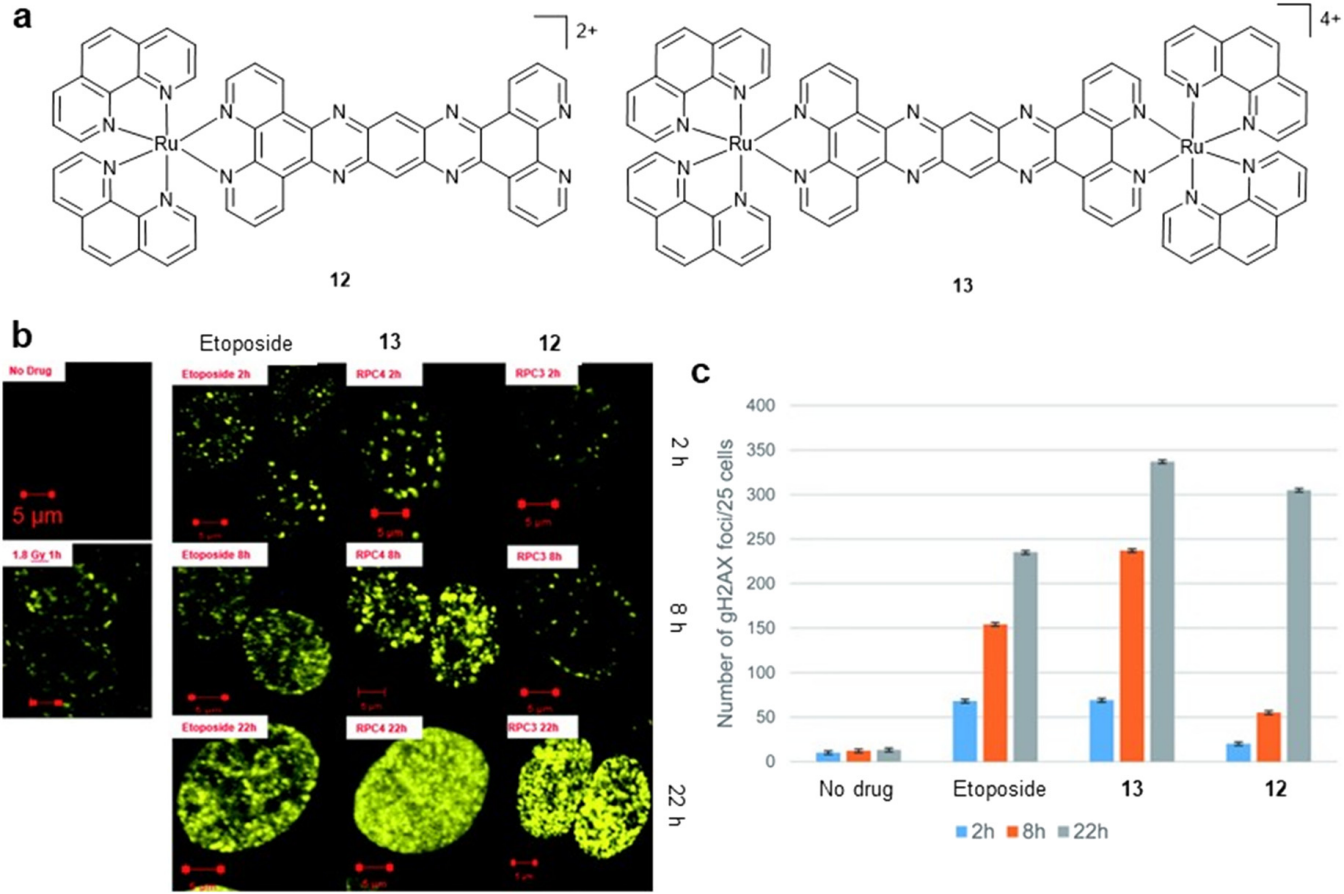
a) Structures of non-photosensitizer DSB-generating RPCs 12 and 13. b) DNA damage as shown by immunofluorescence staining of γH2AX foci in H358 cells treated with IC_50_ values for 13 and 12. The topoisomerase II inhibitor Etoposide employed for comparison. c) Quantification of γH2AX foci for cells treated as in (b). b and c were adapted from ref. 59 under the terms of the CC BY 3.0 license (https://creativecommons.org/licenses/by/3.0/). Published by Royal Society of Chemistry. © The Author 2017.

Although prototypical [Ru(*N^N*)_2_(dppz)]^2+^ complexes bind DNA with high affinities and photocleave DNA *in vitro*, their low bioactivity was a barrier to further progress.^37^ It was realised that to achieve DSB damage in the absence of light, additional ligand functionality was required, either through advanced ligand design or by incorporating metal coordination into established DNA-damaging organic or organometallic frameworks. Macdonnell and co-workers demonstrated that two ruthenium(ii)–tatpp complexes (tatpp = 9,11,20,22-tetraaza tetrapyrido[3,2-*a*:2′3′-*c*:3″,2″-*l*:2‴,3‴]-pentacene), [Ru(phen)_2_(tatpp)]^2+^ (12) and [(Ru(phen)_2_)_2_(tatpp)]^4+^ (13) (Fig. 5a), are effective DNA cleaving agents upon *in situ* reduction by glutathione.^58^13 had previously been shown to bind DNA with high affinity and an intercalative binding mode by the Thomas group^59^ and a similar interaction would be predicted for 12 based on analogous mononuclear RPCs with extended planar polypyridyl ligands. In cells, uptake of 13 in nuclear fractions was detected by graphite furnace atomic absorption spectroscopy and both were cytotoxic towards non-small cell lung cancer H358 cells (IC_50_ values of 13 and 15 μM, respectively).^58^ Moreover, 13 shows enhanced intracellular ROS production and DSB as evidenced by phosphorylated ATM and γH2AX within 2 h of treatment (Fig. 5b and c).^60^ As this is an oxygen-independent mechanism of DNA damage, cytotoxicity remained comparable in hypoxic conditions. A follow-up study found murine acute toxicity of 12 and 13 to be comparable with that for cisplatin and successfully demonstrated tumour growth inhibition in H358 xenografts.^61^ This work was one of the first to demonstrate *in vivo* cancer growth inhibition of systemically administered RPCs that lack labile ligands and also showed that RPCs featuring bioreducible ligands can exhibit inherent cancer selectivity alongside promising hypoxia-selective activity.

**Fig. 6:**
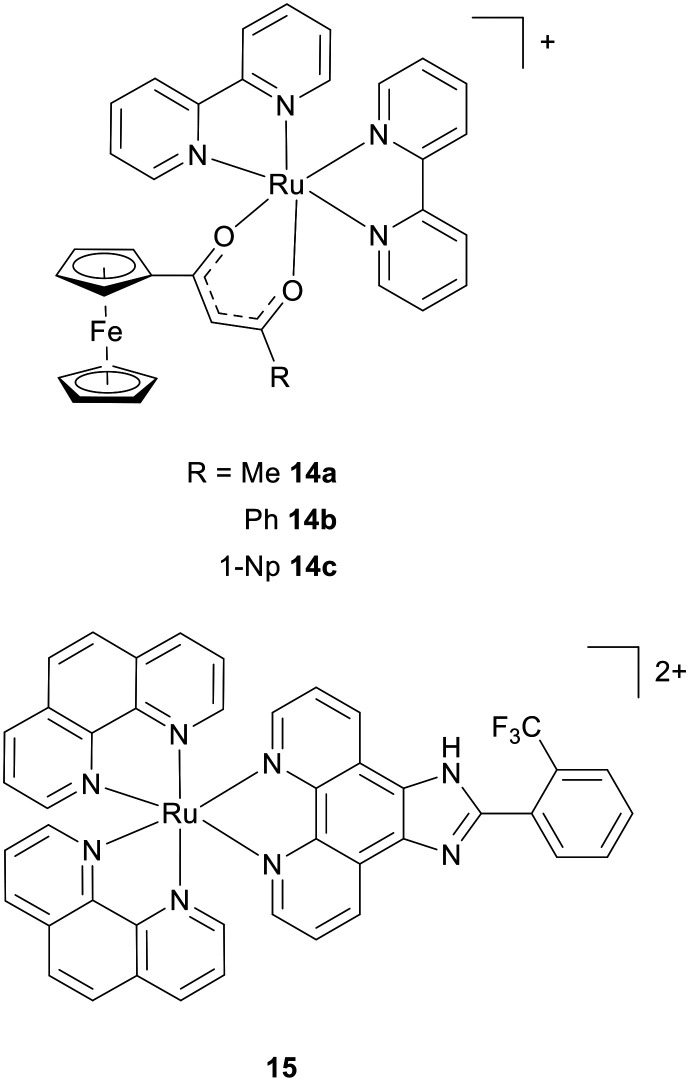
Structures of ferrocene conjugates 14a–c and mitochondrial-targeting agent 15.

Other work includes Allison *et al.* described ferrocene-Ru(bpy)_2_ conjugates that possess high, nanomolar potency towards human cancer cell lines and potencies >40 times greater than cisplatin.^62^ Increased aromaticity of the R substituent on the Fc-acac ligand promoted cytotoxicity while comparison to the *cis*-[Ru(bpy)_2_Cl_2_] precursor and Fc-acac, both of which exhibited low cytotoxicity, indicated the nanomolar potencies of the [Ru(bpy)_2_(Fc-acac)][PF_6_] complexes to be due to the combination of both moieties. Mechanistically, three selected molecules (14a–c, Fig. 6). were found to generate DSBs by Comet assay, and DSB levels correlated with cytotoxicity of this sub-series. Ferrocenes are established ROS generators^63^ and so it is likely that DSB damage is generated through a redox activation mechanism rather than direct DNA binding. Indeed, the incorporation of ferrocenyl groups into scaffolds is a common strategy to isolate new organometallic drug leads.^63^

Finally, numerous anti-cancer drugs, including cisplatin, induce oxidative stress through mitochondrial dysfunction that will contribute towards DNA damage. In the case of cisplatin, mitochondrial ROS generation has even been indicated to provide a measure of sensitivity of ovarian cancer cells to treatment.^64^ Mitochondrial-targeting RPCs that generate ROS and apoptosis are particularly common findings,^65,66^ Selecting one representative example, [Ru(phen)_2_(*o*-CF_3_-PIP)]^2+^ (PIP = (2-(phenyl)imidazo[4,5-*f*][1,10]phenanthroline; 15, Fig. 6). is active towards a small panel of cancer cell lines, with reduced activity towards normal cells.^67^ Disruption of the mitochondrial membrane potential and resultant ROS generation was shown to generate DSB damage, as determined by γH2AX foci assay, in HepG2 human liver cancer cells, ultimately triggering cell death by apoptosis. This study also utilised an experimental liver cancer xenograft model in zebrafish embryos to demonstrate cancer growth inhibition in an animal model. Given that many RPCs are engineered to be more hydrophobic (enhancing cellular uptake) and to incorporate extended polypyridyl ligands (facilitating DNA binding), such a mechanism of action involving mitochondrial dysfunction, ROS generation, and subsequent DSB damage leading to apoptosis, will likely be commonplace.

## DNA replication and transcription inhibitors

**Fig. 7:**
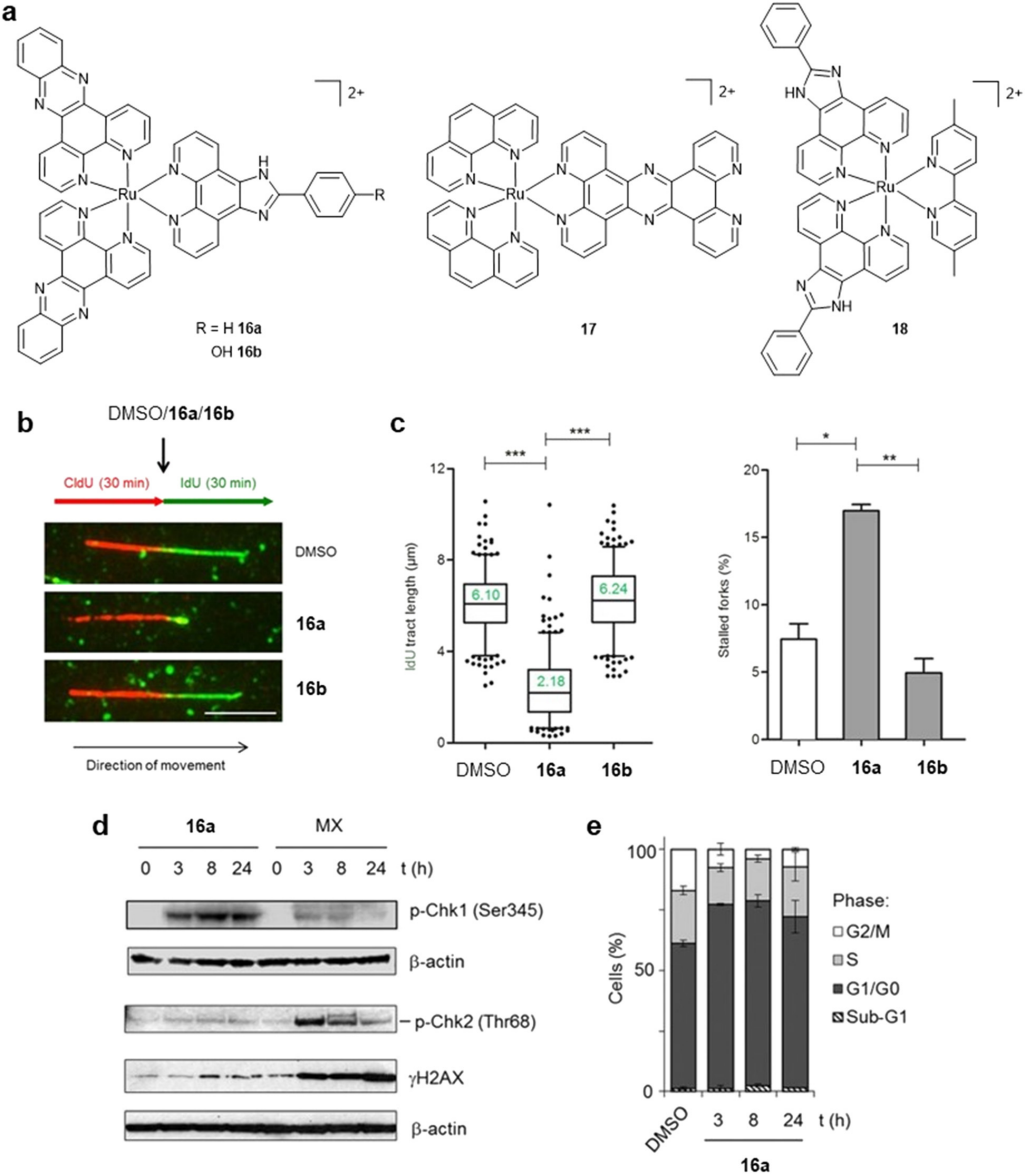
a) Structures of DNA replication inhibitors 16a, 17 and 18. b) Impact of 16a and 16b on replication fork progression in HeLa cells, as determined by DNA fibre assay. c) Quantification of IdU tract length (left) and stalled forks (right) for cells treated as in (b). d) Activation of Chk1 pathway in HeLa cells treated with 16a, as shown by pChk1 (Ser345) generation. Note low Chk2 activation and γH2AX levels. Topoisomerase II inhibitor Mitoxantrone treatment included for comparison. e) Cell-cycle distributions of HeLa cells incubated with 16a. IC_50_ concentration of 16a employed in all experiments. b, c, d and e adapted from ref. 66 under the terms of the CC BY 4.0 license (https://creativecommons.org/licenses/by/4.0/). Published by Springer Nature Limited. © The Author 2016.

Advances in biological techniques or assay design can also facilitate mechanistic understanding. In 2016, the Vallis group employed DNA fibre assays, a single-molecule imaging technique able to directly monitor DNA replication dynamics, to demonstrate that the Ru(ii) metallo-intercalator [Ru(dppz)_2_(PIP)]^2+^ (16a, Fig. 7a) slows DNA replication fork progression and increases the number of stalled forks (Fig. 7b and c).^68^ This represented the first direct evidence that a substitutionally inert RPC functions as a DNA replication inhibitor in cells. Fork stalling by 16a was accompanied by ATR–Chk1 pathway activation, however, limited activation of Chk2 and low pH2AX expression was observed, indicating that fork stalling occurs without DSB generation (Fig. 7d). Likely due to this, apoptosis was not triggered and instead G1 cell-cycle arrest was apparent (Fig. 7e). These results contrasted with other classes of replication inhibitors, such as topoisomerase II inhibitors. Subsequent work found a similar impact of [Ru(phen)_2_(tpphz)]^2+^ (17, Fig. 7a) on replication fork speed in OE21 oesophageal cancer cells, however, in this case, activation of Chk2 and pH2AX along with G2/M arrest was consistent with DSB generation resulting from the collapse of stalled forks.^69^ DNA damage persevered after complex removal and cell fate was mixed, with evidence of a senescence phenotype and, most intriguingly, a defect in mitosis-spindle attachment failure. Reduced cross-resistance towards 17 in A2780/CP70 cells compared to cisplatin was found.^70^ Structural modification of 17 demonstrated that while groove binding derivatives experienced a reduction in cytotoxicity potency, there was no impact on resultant cross-resistance in platinum-resistant A2780CIS cancer cells, suggesting that DNA intercalation is *not* required to bypass platinum resistance.^71^ Recently, the Gill group identified that [Ru(PIP)_2_(dmb)]^2+^ (dmb =5,5′-dimethyl-bpy; 18, Fig. 7a) similarly slows fork progression.^72^ From these results, key structural requirements for replication inhibition would appear to be the presence of multiple intercalative units and/or an extended intercalating ligand(s) coordinated to the central Ru(ii) ion. However, clear differences in the DDR responses to 16a and 17, most notably that 16a does not trigger DSB pathway activation, would indicate that there is not a general DDR response to RPC intercalation. Based on these observations, it seems plausible that RPC intercalators generate lesions that present a physical barrier to slow or halt fork progression, although the structural and biological factors that govern whether stalled forks are stabilised (resulting in cell-cycle arrest) or collapse (generating DSB damage and triggering apoptosis) remain to be elucidated.

**Fig. 8:**
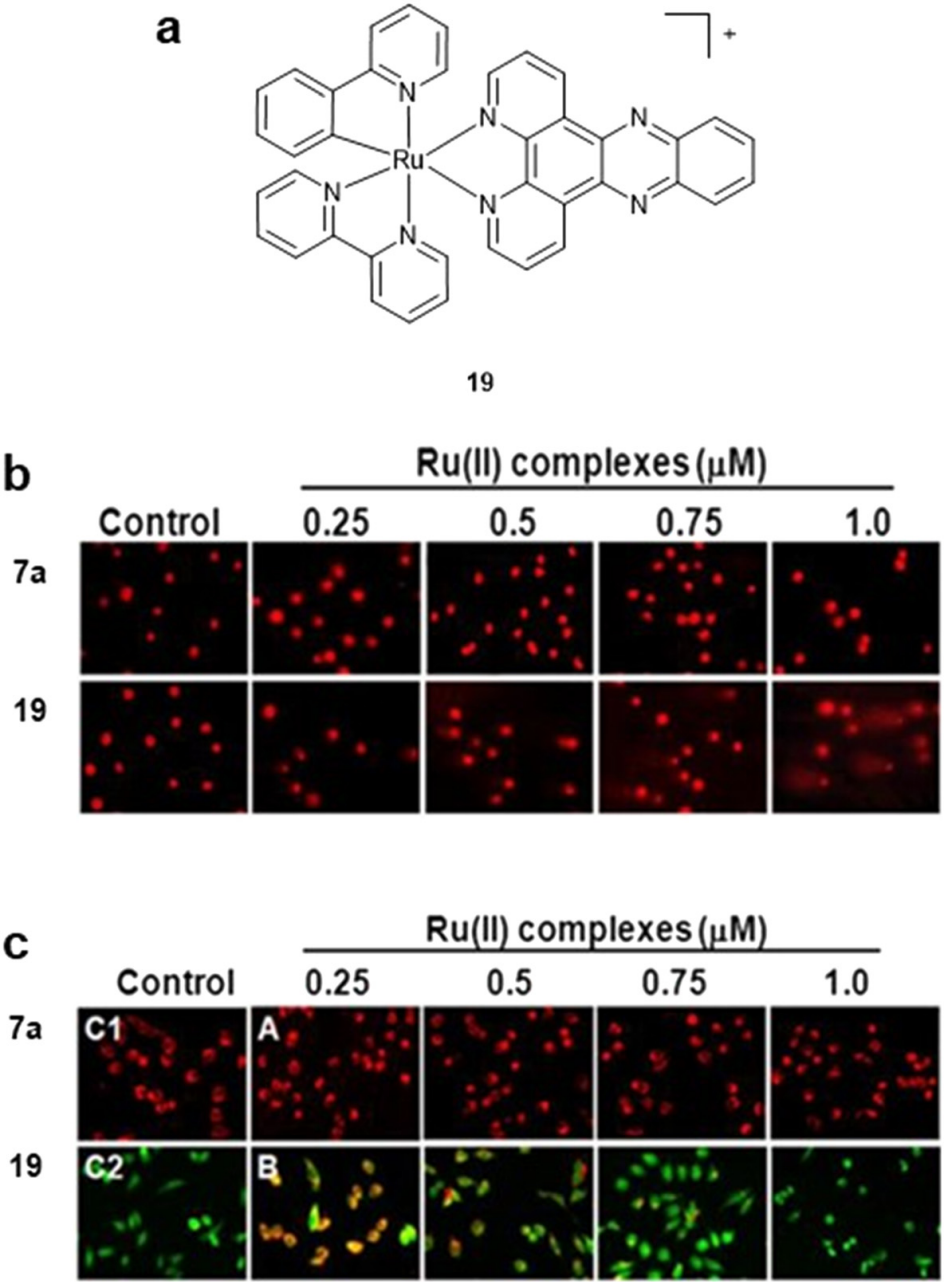
a) Structures of cyclometalated monocationic Ru(ii) complex 19. b) Chromosomal DNA strand breaks in HeLa cells induced by 19 as detected by comet assay. c) Compromised mitochondrial membrane potential in in HeLa cells treated with 19 (green fluorescence), as detected by JC-1 assay. Treatment with 7a included for comparison. b and c reprinted (adapted) with permission from ref. 73. © 2014 American Chemical Society.

In addition to DNA replication, RPCs have also been reported to block transcription, where RPCs with high DNA binding affinities can block RNA polymerases binding to the template DNA.^73,74^ The Chao group reported the cyclometalated Ru(ii) complex, [Ru(bpy)(phpy)(dppz)]^+^ (19, Fig. 8a) to disrupt binding of the NF-κB transcription factor to DNA in cell-free conditions.^75^ In cells, 19 localised primarily in the nucleus and suppressed DNA replication as shown by EdU assay. Cytotoxic potencies greater than cisplatin were observed, including in a 3D HeLa multicellular tumour spheroid model, and there was no cross-resistance in cisplatin-resistant A549 and A549/CDDP lung carcinoma cell lines. Mechanistically, while DNA damage was indicated by comet assay, 19 also disrupted mitochondrial membrane potential determined by JC-1 assay (Fig. 8b and c). This is a similar mechanism as for the mitochondrial-targeting complex 15 above and correlates with an increase in hydrophobicity promoting a mitochondrial-targeting mechanism of action. Nonetheless, this work is significant as one of the rare examples of a bio-active Ru(dppz) complex and the direct comparison with 7a emphasises the high importance of charge in overall complex design.

## Adduct formation and photo-activated chemotherapy (PACT)

**Fig. 9:**
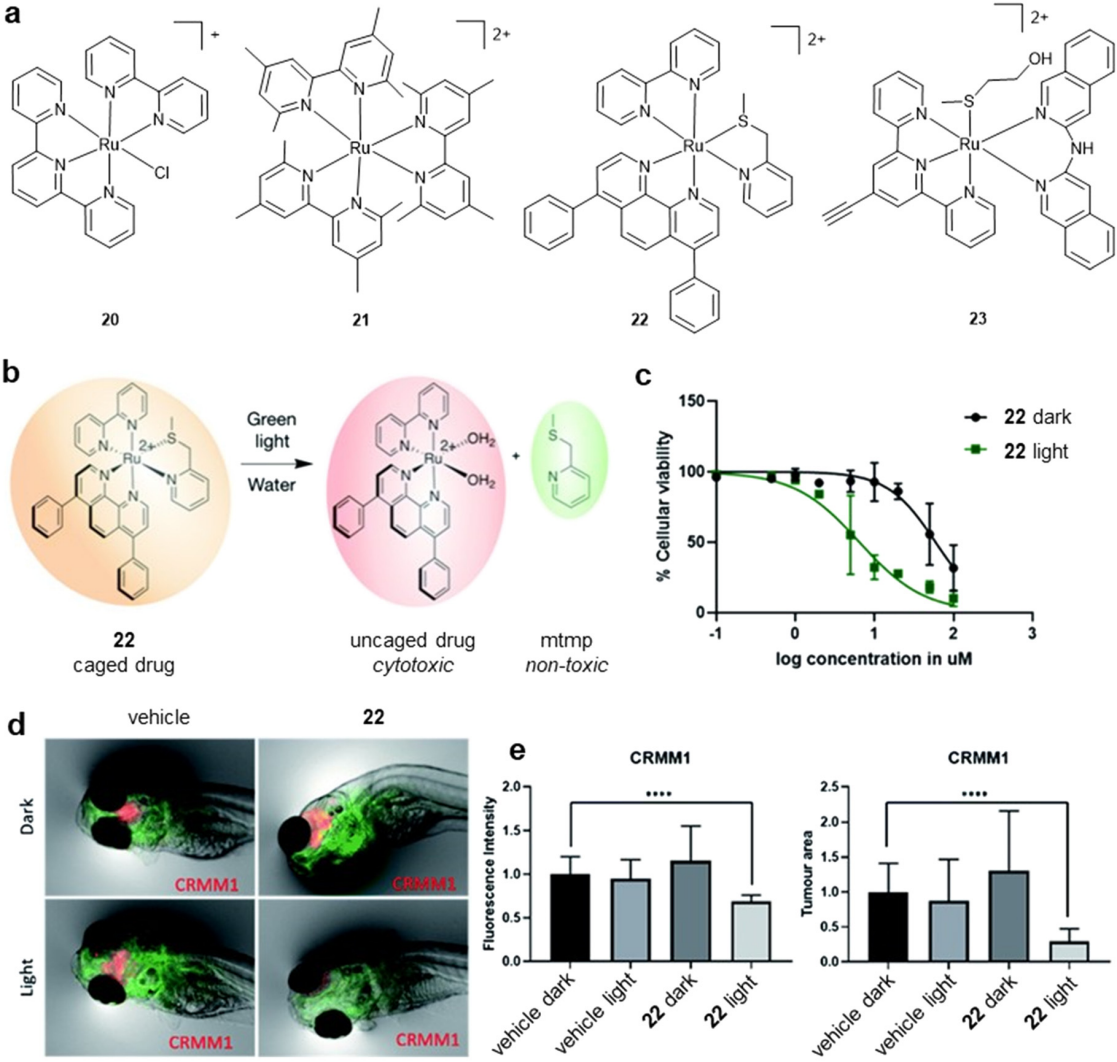
a) Structures of RPCs implied in DNA adduct formation. b) Schematic of the activation mechanism of the RPC PACT complex 22. c) Dose–response curves for A549 cells treated with 22. Cells were either irradiated with green light (520 nm, 15 min, 21 mW cm^−2^, 19 J cm^−2^) 24 h after treatment (green data points) or left in the dark (black data points). d and e) Efficacy of the 22 in a zebrafish orthotopic model of conjunctival melanoma. b–e were adapted from ref. 81 under the terms of the CC BY 3.0 license (https://creativecommons.org/licenses/by/3.0/). Published by Royal Society of Chemistry. © The Author 2022.

As discussed, cisplatin is able to form platinum-DNA adducts and, due to possessing two labile groups, generate intra- and interstrand DNA crosslinks or even DNA-protein crosslinks. Although the majority of RPCs are designed for reversible (non-covalent) binding, RPCs containing at least one R–Cl bond (or other labile ligand available for substitution) would likewise be predicted to form a coordinate bond with the base pairs of DNA. This concept was first demonstrated by Barton and Lolis^76^ and further explored by Nováková *et al.*^77^ This latter study presented evidence that all Ru(ii) complexes coordinate DNA at multiple sites (with a preference for guanine residues) and terminate DNA replication in cell-free conditions. Moreover, this study found one complex, mer-Ru(tpy)Cl_3_ (tpy = 2,2′:6′,2″-terpyridine; 20, Fig. 9a), to form DNA cross-links and to demonstrate activity in L1210 leukaemia cells. Despite these early studies, comparatively few RPCs relative to Pt(ii) and Ru(arene) systems have been shown to form DNA adducts and exhibit bioactivity. This may partly reflect the strong emphasis on non-covalent DNA binding interactions of RPCs, but also the inherent steric constraints imposed by ancillary polypyridyl ligands, which disfavour the formation of Ru–DNA coordination bonds. Poor aqueous solubility is an additional barrier for the use of neutral or mono-cationic species common for Ru(ii) chloride complexes.

A promising alternative approach has been the utilisation of RPCs in the design of light-activated pro-drugs within photo-activated chemotherapy (PACT). This exploits the ability of the Ru(ii) centre to undergo photoinduced ligand substitution, whereby light-triggered loss of a polypyridyl ligand “uncages” a coordinatively active Ru(ii) species capable of directly binding or “ruthenating” DNA. PACT offers a distinct advantage over the related PDT as it does not require oxygen so retains effectiveness in hypoxic conditions.^78^ Work by the Glazer group has explored the intracellular photoactivated release of polypyridyl ligands in detail.^79–81^ A good example is the photoejecting compound 21 (Fig. 9a), which is capable of inducing *in vitro* DNA strand breaks or covalent adducts upon light activation.^82^ Although intracellular ruthenation or resultant DNA damage was not characterised, 21 was able to completely abrogate new protein synthesis in HEK293 T-Rex-dendra2 cells at 30 μM. Another leading example is presented by the Bonnet group who designed a novel trisheteroleptic ruthenium complex [Ru(DIP)(bpy)(mtmp)]^2+^ (mtmp = 2-methylthiomethylpyridine; 22, Fig. 9a).^83^ Upon exposure to green light, the non-toxic mtmp ligand is released, generating the uncaged active Ru(ii) group that is theorised to ruthenate DNA (Fig. 9b). Cytotoxicity as a result of photosubstitution was demonstrated in several cancer cell lines and zebrafish embryo tumour models (Fig. 9c–e). Supporting the design hypothesis, 22 was able to photocleave DNA in cell-free conditions. However, no investigation of cellular DNA damage induced by the active Ru(ii) species was performed.

In addition to the Ru(ii) centre, the released ligand may possess independent bio-activity. Busemann *et al.* designed a photo-activated chemotherapy complex, [Ru(HCC-tpy)(i-Hdiqa)(Hmte)](PF_6_)_2_ (23, Fig. 9a) (HCC-tpy = 4′-ethynyl-2,2′:6′,2″-terpyridine, i-biq = 3,3′-biisoquinoline i-Hdiqa = di(isoquinolin-3-yl)amine, Hmte = 2-(methylthio)ethanol).^84^ This complex was designed for loss of the Hmte ligand and found to be cytotoxic towards A547 lung cancer cells (IC_50_ = 29 μM); an effect further enhanced by light activation (IC_50_ = 7 μM). 5-Vinyl-2′-deoxyuridine DNA (VdU) labelling was consistent with the inhibition of DNA synthesis by light-activation. This concept can be utilised to employ released ligands that possess complementary mechanisms of activity to Ru adduct formation. Zamora *et al.* were able to demonstrate this concept by coordinating P450 inhibitors to a Ru(bpy)_2_ centre, where three Ru(ii) complexes all demonstrated light-activated enzyme inhibition.^85^ While encouraging, these studies show a high reliance on cell-free DNA cleavage assays and it remains an unverified hypothesis that the uncaged Ru moiety can induce cellular DNA damage, what form such lesions might take and how they influence cellular responses. Such mechanistic understanding would be of relevance considering that current RPCs within PACT display modest phototoxicity indices (PI), typically <10, which may be compared to PDT photosensitizers where PIs of >100 or greater are commonly encountered.^83^

## DNA condensation

**Fig. 10:**
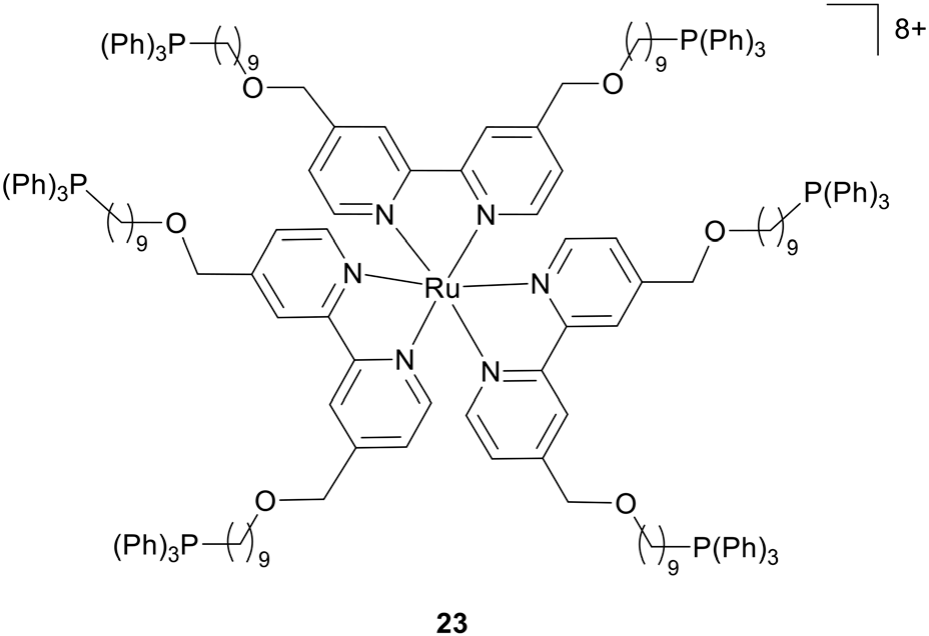
Structure of RPC 23 shown to condense DNA.

RPCs can also act to condense DNA, which is typically facilitated by significantly larger structures.^86,87^ This is best illustrated by the Chao group who designed four Ru(ii) complexes with high positive charges of +8 valence (Fig. 10).^88^ These structures are able to promote interactions reminiscent of histone-mediated DNA packaging. Cellular uptake was improved by alkyl chain length, with nuclear accumulation greatest (approximately 60.4%) for complex 23. Cytotoxicity testing against several tumour and drug-resistant cell lines (including A375, MDA-MB-231, SW620, A549, A549/DDP, SGC7901, and SGC7901/DDP) indicated 23 was effective against drug-resistant and parental tumour cells. *In vitro* inhibition of DNA replication and binding of the NF-Kβ transcription factor to DNA was shown. RNA-seq analysis of A549/DDP cells treated with 23 revealed that genes related to DNA polymerase, DNA helicase, and RNA polymerase II were downregulated, suggesting that replication and transcription were impacted by intracellular DNA condensation. A deficiency in this study is that DNA damage or DDR signalling was not examined in cells and that no clear cell death pathway was determined. Thus, at the time of writing, it is unknown how cancer growth inhibition is achieved by this DNA binding interaction.

## Emerging concepts

### Non-duplex structures

**Fig. 11:**
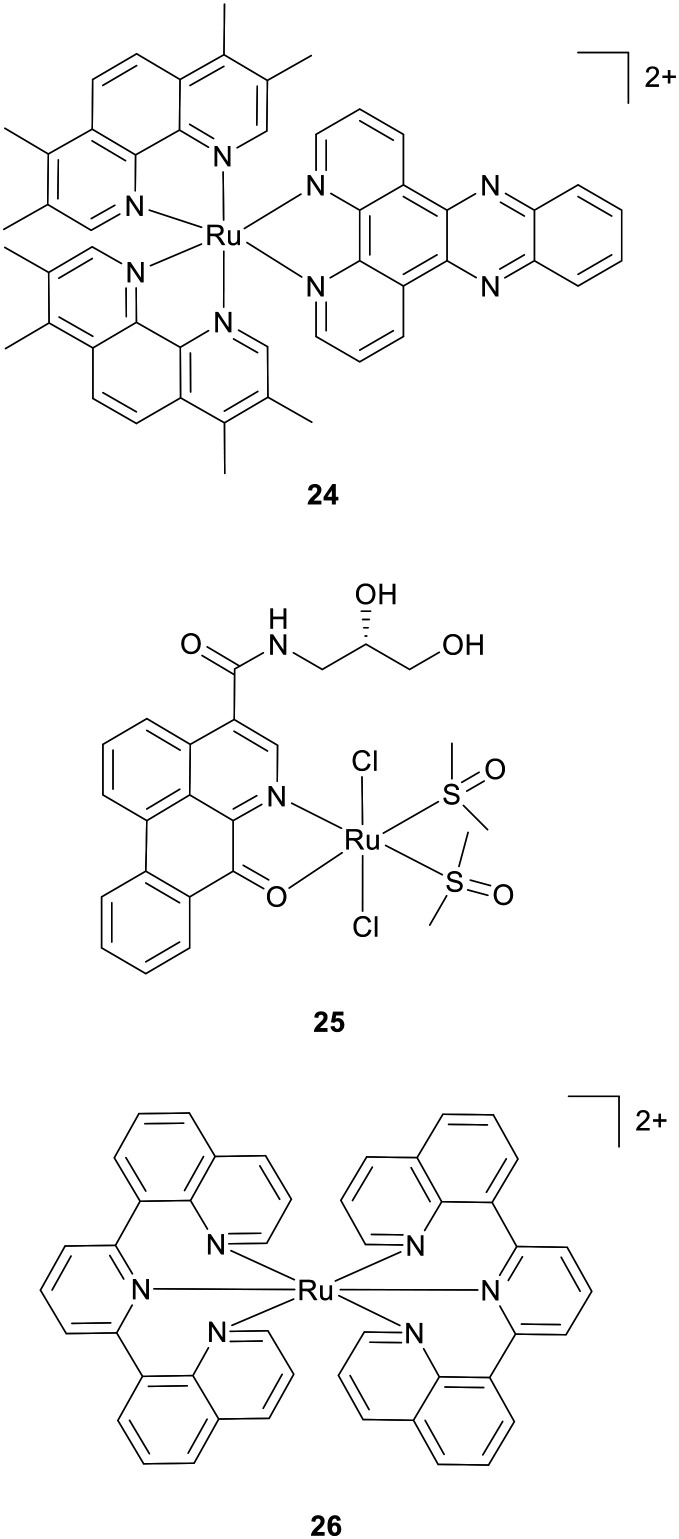
Structures of RPCs that interact with non-canonical DNA, including mismatch DNA (complex 24), G4 quadruplex DNA (complex 25) and i-motifs (complex 26).

In addition to duplex DNA binding, ligand design and selection can influence selectivity for specific sequences of DNA. An example of this is the design of mismatch-interactive RPCs where the use of bulky methylated ancillary ligands decreases affinity for well-matched DNA whilst preserving the ability to interact with larger, less stabilised mismatch sites.^89,90^ This can achieve distinct binding modes and resultant structural changes compared to conventional duplex binding, for example the metallo-insertion of 7a at a mismatch base results in the ejection of a base pair.^91^ Mismatch-interactive complexes such as [Ru(tmp)_2_(dppz)]^2+^ (tmp = 3,4,7,8-tetramethyl-1,10-phenanthroline; 24, Fig. 11) have shown enhanced cytotoxicity towards MMR-deficient cancer cell lines.^92^ Although the relationship between MMR-deficiency and cellular DNA damage in response to RPC treatment remains to be delineated, the Barton group have shown that a rhodium metalloinsertor generated a DNA lesion in MMR-deficient cells,^93^ consistent with a DNA base pair mismatch as the cellular target.^94^

Alternative DNA structures have also been targeted, most notably G-quadruplex-DNA (G4-DNA). Chen *et al.* prepared three water-soluble ruthenium(ii) complexes with chiral 4-(2,3-dihydroxypropyl)-formamide oxoaporphine (FOA) ligands. High chiral sensitivity was found, with complex 25 (LC-003) *cis*-[RuCl_2_(*S*-(−)-FOA)(DMSO)_2_] (Fig. 11) stabilising telomeric and G4-DNA, inhibiting telomerase and promoting c-myc in cells, ultimately preventing cell proliferation by induced cell senescence and apoptosis.^95^ Potency towards cancer cell lines was comparable to cisplatin and ranged from IC_50_ concentrations 7.4 to 16.2 μM. However, and in contrast to cisplatin, a reduced activity towards HL-7702 normal cells was found (IC50 = 69.2 μM). Encouraging tumour growth inhibition in a BEL-7402 xenograft mouse model along with improved *in vivo* safety to cisplatin was also shown. Similar to G4 quadruplexes, complementary C-rich sequences can fold into four stranded i-motifs and RPCs such as [Ru(bqp)_2_]^2+^ (bqp = 2,6-bi(quinolin-8-yl)pyridine; 26, Fig. 11) hold early promise as i-motif detecting agents.^96^ As work is only beginning to reveal the impact small molecules that bind quadruplexes may have at the cellular level, which can range from localised DNA damage to gene expression,^97,98^ and even less is known about i-mofits, it seems likely that RPCs will find application as molecular tools towards such ambitions.

### Combination with DDR inhibitors

Given this understanding of DDR activation by an RPC, a logical next step is to assess whether drug synergy can be achieved using targeted inhibitors of signalling proteins within the relevant pathway. This can enhance the potency of the inhibitor, potentially decreasing active dose required, expand activity to include a greater range of cancer types, and combat ongoing challenges such as resistance, which can be problematic for targeted cancer therapeutics considering the heterogeneous nature of the disease.

PARP inhibitors have made encouraging clinical progression where they are effective as single-agents towards BRCA-deficient cancers. However, researchers have also examined their ability to be combined with other treatment modalities, including DNA-damaging chemotherapy and metallodrugs.^99,100^ Such a strategy can enhance PARPi potency, combat drug resistance and expand the range of cancers to include BRCA-proficient sub-types.^101^ The aforementioned replication inhibitor 16a synergises with Olaparib, where treatment of MDA-MB-231 or MCF7 cells renders both cell lines hypersensitive to Olaparib.^102^ Synergy is explained by PARP inhibition resulting in the collapse of 16a-stalled forks, generating DSB damage which triggers cell death by apoptosis.

**Fig. 12:**
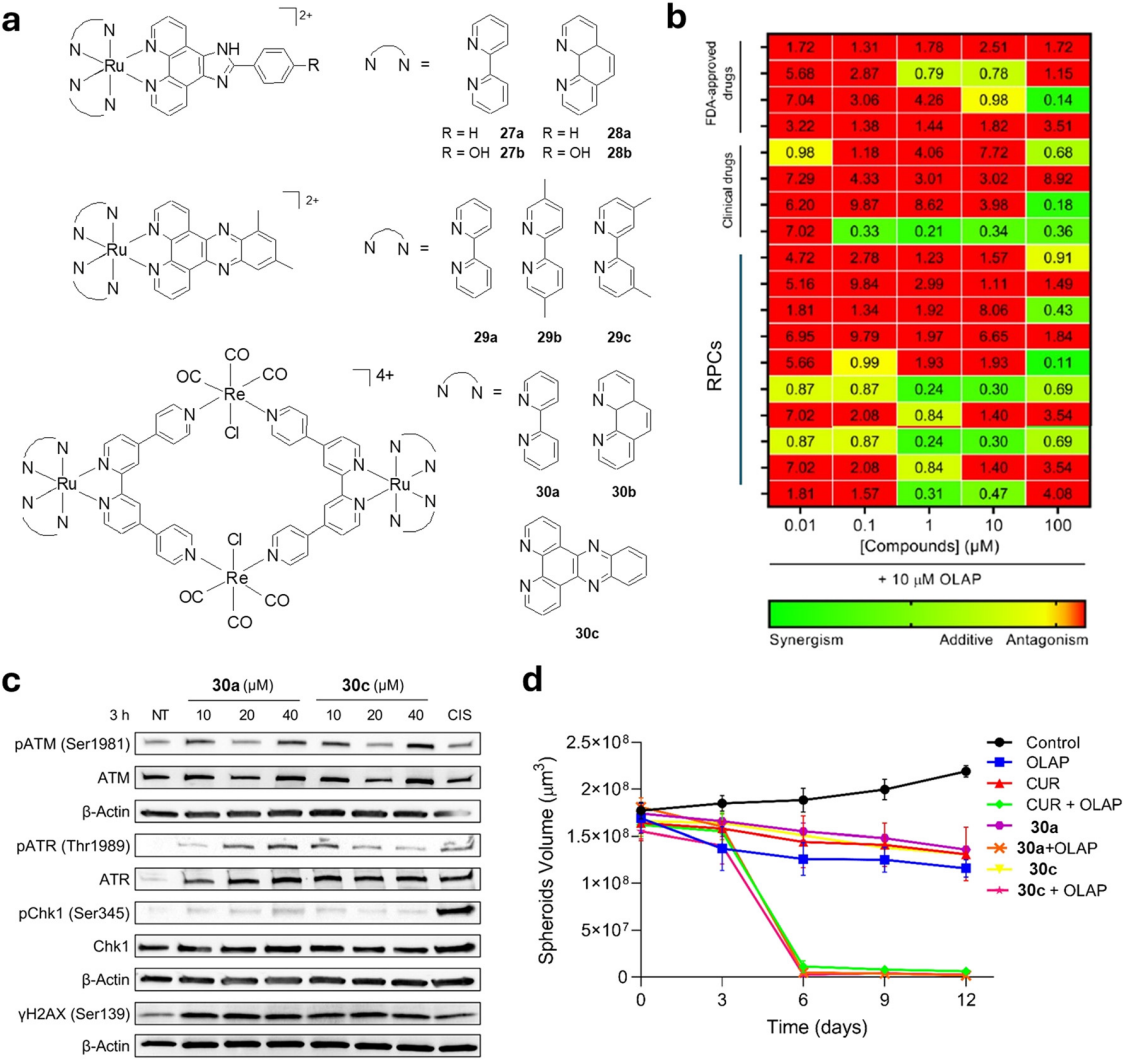
a) Structures of DNA-binding RPCs featured within a chemically diverse “micro library”. b) Results of screening for Olaparib synergy. c) Activation of ATM and ATR pathways in response to treatment with 30a or 30c. d) Impact of 30a and 30c on MDA-MB-231 cell spheroid growth without and with Olaparib co-treatment. Data were adapted from ref. 114 under the terms of the CC BY 4.0 license (https://creativecommons.org/licenses/by/4.0/). Published by the American Chemical Society. © The Author 2023.

Synergy screens with specific DDR inhibitors can isolate molecules that generate DNA damage, even if this is not their primary mechanism of activion.^103^ The Gill, Thomas and Ahmad groups applied this to assemble a small library of RPCs (27a–30c, Fig. 12a). These were combined with FDA-approved drugs and established DNA-damaging agents to form a chemically diverse “micro library” that was screened for synergy with Olaparib (Fig. 12b).^104^ This isolated two RuRe metallomacrocycles 30a and 30c as the strongest hits and activation of ATM and ATR indicated both SSB and DSB damage was generated by each complex (Fig. 12c). Mechanistic studies into drug synergy found co-treatment with Olaparib to be accompanied by enhanced DSB formation and apoptosis compared to either agent in isolation, consistent the conversion of 30a and 30c-generated SSB damage to DSBs as a result of PARP inhibition, and drug synergy was retained in 3D spheroid models (Fig. 12d).

Other DDR inhibitors represent strong candidates to explore in synergy studies. Of particular relevance are ATR inhibitors (ATRi), which are of great current interest in cancer treatment and are especially effective within combination therapy.^105^ Based on this, the Ahmad group successfully found 16a and a series of nitro-substituted derivatives synergise with the ATRi berzosertib or ceralasertib in multiple cancer cell lines.^106^ This work also showed that the nitro-substituted derivative [Ru(dppz)_2_(*p*-NPIP)]^2+^ (where NPIP = 2-(nitrophenyl)imidazo[4,5-*f*][1,10]phenanthroline) showed an elevated DSB damage response compared to the parent molecule 16a, thereby demonstrating the ability of nitro-substitution to alter specific DDR pathway activation.

Further exploration of DNA-targeting RPCs in drug synergy studies would expand these concepts and promising combinations would be particularly well-suited to a drug delivery approach as this may provide molecular targeting or localised release alongside dual delivery.^107^ Furthermore, based on the presumed generation of high DSB damage, RPCs employed in PDT would also represent strong candidates for combination with DDR inhibitors, where addition of the inhibitor would be predicted to lower the active dose of light required for phototoxicity and enhance phototoxicity indices.

### Safety and genotoxicity

As for any DNA-targeting small molecule, including DDR inhibitors, there will naturally be concerns about safety and potential mutagenic or genotoxic effects. This is going to be in an adduct-related fashion: unrepaired DSBs in particular can result in loss of genetic information, chromosomal aberrations, or cell death.^108^ However, it is also the case that the precise location of a single DSB within the genome may impact the cellular response.^109^ For non-covalent interactions, despite a popular conception that intercalators such as ethidium bromide are potent mutagens, a systematic search of the literature by Ferguson and Denny in 2007 found there to be very little evidence supporting this.^110^ Instead, they concluded that intercalation primarily acts to anchor a molecule to DNA and mutation effects are dominated by secondary effects such as topoisomerase II inhibition. As “simple intercalators”, many of the RPCs featured within this review would therefore not be predicted to possess significant mutagenicity, although it is fair to acknowledge that this requires further investigation, particularly as more complexes progress towards clinical applications.

A small number of studies have attempted to link DNA damage to mutagenicity or genotoxicity. Employing a zebrafish embryo model, treatment with the replication inhibitor 16a showed few morphological changes of the zebrafish embryos, consistent with low DSB levels.^111^ The HPRT-forward mutation assay in V79 Chinese hamster cells showed 17 to generate a mutation frequency approximately half that of cisplatin,^69^ consistent with both molecules eliciting a DSB damage response and generating the most genotoxic lesion. In addition to this, lethal or safe operating doses for a few candidates have been examined in mouse models for subsequent xenograft studies.^61,95^

### Limitations of current biological models

It is evident that most of the studies described above rely on 2D cellular models. The limitations of these systems are well established: cells are grown as unrealistic monolayers that fail to recapitulate the *in vivo* tumour architecture, cellular behaviour (including signalling pathways) is altered relative to native tissue and key features of the tumour microenvironment, such as stromal interactions and physiological gradients (*e.g.* hypoxia and nutrient availability), are absent. In addition, drug exposure in 2D cultures is effectively uniform, which can lead to an overestimation of therapeutic efficacy.

3D models offer improved physiological relevance, yet important limitations remain. These systems typically lack true heterogeneity and functional vasculature, and their implementation is associated with increased cost and technical complexity. Furthermore, in both 2D and 3D cultures, tissue culture plastic is commonly treated to impart a net negative surface charge. As a result, RPCs may adsorb to these surfaces, reducing their effective concentration in the culture medium and potentially forming a surface-bound layer that alters cell adhesion and motility.

It is also notable that many studies employ relatively generic cancer cell lines and focus primarily on cytotoxicity, often benchmarked against cisplatin, with subsequent target validation used to support *in vitro* binding data. While this strategy has been valuable for identifying early cytotoxic leads, including compounds that target DNA, advances in cancer genetics and tools for precise control of gene expression have shifted modern drug discovery towards identifying specific genetic determinants of drug activity and resistance. This approach enables the stratification of therapeutic candidates towards defined cancer subtypes. Applying such strategies to RPCs would enhance their biological relevance and provide deeper mechanistic insight.

Finally, there is little known on the pharmacodynamics or pharmacokinetics of RPCs. While historical work by Dwyer and coworkers in the 1950s indicated radiolabelled [Ru(bpy)_3_]^2+^ to pass through mice models intact,^112^ a more recent biodistribution study in mice showed a radiolabelled RPC accumulates primarily in the liver,^113^ and the safety profile of TLD1433 has been assessed in a phase 1 trial,^114^ it is unknown how structural modifications featured in this review would impact these properties. Therefore, examining RPC metabolism in mammalian models to identify common metabolites is an area of immediate importance. Along with this aspect, preclinical studies to establish therapeutic windows are also required which is of particular importance considering that DNA is the target and the safety considerations outlined above.

## Conclusions and outlook

The DNA-binding properties of ruthenium(ii) polypyridyl complexes continue to attract interest owing to their well-developed synthetic chemistry and defined molecular geometries, combined with distinctive photophysical responses upon binding. It is now well established that these complexes can interact with DNA through a range of binding modes and give rise to diverse biological outcomes, with DDR signalling representing a key mechanistic link between binding interactions and downstream cellular effects. Notable differences between ruthenium polypyridyl complexes and cisplatin are evident, including that Ru–DNA adducts are more resistant to removal, which may contribute to reduced cross-resistance in cisplatin-resistant cancers. Furthermore, several functional modalities are now well characterised: purely intercalative systems can inhibit DNA replication without inducing genotoxic double-strand breaks, redox-active complexes can act as catalytic DNA-cleavage agents following reduction by glutathione, and photodynamic therapy candidates enable localised reactive oxygen species generation or photo-uncaging of bioactive Ru(ii) species.

At the same time, important uncertainties remain. Traditional structure–activity relationships in this area are still somewhat limited and, as cellular uptake and nuclear targeting are highly structure-sensitive, small ligand modifications can profoundly alter biological outcomes. Consequently, many studies focus on single complexes or small series, which makes it difficult to establish general design principles. The role of chirality also remains underexplored: although resolved Λ and Δ enantiomers are known to interact differently with DNA in cell-free systems,^115^ and studies examining chirality on cellular uptake and cytotoxicity have been undertaken,^116^ most biological studies employ racemic mixtures and more widespread use of resolved enantiomers would greatly expand our understanding of the nature of DNA damage generated. Similarly, while DDR activation is frequently used for target validation, the contribution of specific DNA repair pathways (*e.g.* base excision repair, nucleotide excision repair, homologous recombination, and non-homologous end joining) has not yet been examined in detail. A final consideration is the limited pre-clinical evaluation at present, which would facilitate pharmacological assessment and establish therapeutically relevant dosing windows.

Looking ahead, several research directions appear particularly promising. There remains clear scope for medicinal chemistry optimisation through systematic ligand modification, with the aim of improving potency while retaining mechanistic activity. More widespread use of resolved enantiomers would provide valuable insight into chiral effects on DNA binding and damage. Improved cancer selectivity may be achieved through targeting non-canonical DNA structures, combination therapies involving DDR inhibitors such as PARP inhibitors, or incorporation into drug-delivery platforms. Furthermore, a more comprehensive evaluation of DNA repair responses could not only enhance mechanistic understanding but also serve as a predictor of therapeutic response, particularly in cancers with defined repair deficiencies. Addressing these areas, alongside expanded pharmacological and toxicological studies, will be essential for advancing RPCs towards clinically relevant metal-based DNA-targeting agents.

## Conflicts of interest

There is no conflict of interest to declare.

## Data Availability

No primary research results, software or code have been included and no new data were generated or analysed as part of this review.
